# Cycloheximide can distort measurements of mRNA levels and translation efficiency

**DOI:** 10.1093/nar/gkz205

**Published:** 2019-03-27

**Authors:** Daniel A Santos, Lei Shi, Benjamin P Tu, Jonathan S Weissman

**Affiliations:** 1 Department of Cellular and Molecular Pharmacology, University of California San Francisco, San Francisco, CA 94158, USA; 2 Department of Biochemistry, University of Texas Southwestern Medical Center, Dallas, TX 75390-9038, USA; 3 Howard Hughes Medical Institute, University of California San Francisco, San Francisco, CA 94158, USA

## Abstract

Regulation of the efficiency with which an mRNA is translated into proteins represents a key mechanism for controlling gene expression. Such regulation impacts the number of actively translating ribosomes per mRNA molecule, referred to as translation efficiency (TE), which can be monitored using ribosome profiling and RNA-seq, or by evaluating the position of an mRNA in a polysome gradient. Here we show that in budding yeast, under nutrient limiting conditions, the commonly used translation inhibitor cycloheximide induces rapid transcriptional upregulation of hundreds of genes involved in ribosome biogenesis. Cycloheximide also prevents translation of these newly transcribed messages, leading to an apparent drop in TE of these genes under conditions that include key transitions during the yeast metabolic cycle, meiosis, and amino acid starvation; however, this effect is abolished when cycloheximide pretreatment is omitted. This response requires TORC1 signaling, and is modulated by the genetic background as well as the vehicle used to deliver the drug. The present work highlights an important caveat to the use of translation inhibitors when measuring TE or mRNA levels, and will hopefully aid in future experimental design as well as interpretation of prior results.

## INTRODUCTION

The development of ribosome profiling has broadly enabled genome-wide analyses of active translation *in vivo* (1). The technique is based on deep sequencing of ribosome-protected mRNA fragments, or ribosome footprints, providing a quantitative snapshot of ribosome positions along mRNAs at single nucleotide resolution. When combined with traditional RNA-seq, it is possible to determine the ratio of ribosome footprints to the number of mRNA molecules for a given gene, thereby providing a measure of translation efficiency (TE) for that gene. In contrast to absolute measures of translation rates provided by ribosome profiling, TE measurements require accurate quantitation of both translation rates and mRNA abundances. TE is partially governed by intrinsic features of an mRNA such as its sequence and structure (2), but TE can also vary dynamically as a regulatory strategy. Two notable examples in budding yeast are the transcription factors Hac1 and Gcn4, whose mRNAs rapidly transition from low to high TE states when cells encounter specific stresses. Interestingly, the mechanisms driving the TE switch for these factors are completely distinct: *HAC1* mRNA is spliced by the ER-resident protein Ire1 to remove inhibitory secondary structures during the unfolded protein response (3), and *GCN4* utilizes a series of short upstream open reading frames (uORFs) in its 5′ untranslated region (UTR) that sequester ribosomes away from the main ORF in the absence of stress-induced eIF2α phosphorylation (4).

While these well-studied examples highlight the complexities of translational regulation, other instances of dynamic TE switching remain poorly understood. We initially set out to identify and characterize TE regulation during the yeast metabolic cycle (YMC), a process involving synchronized growth with well-established, coordinated gene expression changes. The YMC is initiated by culturing *Saccharomyces cerevisiae* in a chemostat with limiting glucose to control growth rate and maintain constant culture density. Under these conditions, cells grow and divide in sync, and a redox cycle is established with periodic bursts of respiration resulting in abrupt decreases in dissolved oxygen (5). Gene expression profiling has identified three distinct phases of gene expression as part of this cycle—termed the Reductive Building (RB), Reductive Charging (RC), and Oxidative (OX) phases—with greater than 50% of the overall transcriptome exhibiting variable expression (6).

Using ribosome profiling and RNA-seq, we observed that the majority of genes involved in ribosome biogenesis (commonly referred to as *ribi* genes (7)) appear to dynamically shift their TE state between the RC and OX phases of the metabolic cycle. Moreover, analysis of previous ribosome profiling studies suggests similarly large TE changes for ribi genes during amino acid starvation (8) and meiosis (9), initially suggesting a shared mechanism. However, these experiments were all conducted by treating cells with the translation elongation inhibitor cycloheximide (CHX) prior to harvesting, and upon repeating the amino acid starvation experiment without CHX, we show that TEs of ribi genes remain unchanged. We additionally demonstrate that CHX causes rapid accumulation of ribi transcripts in a TORC1-dependent manner, and the magnitude of the response to CHX depends on strain genotype and the choice of drug vehicle. Thus, CHX distorts measures of TE by causing rapid transcriptional changes which creates a new pool of untranslated mRNAs. These results underscore the caution that needs to be taken when using CHX as an experimental tool.

## MATERIALS AND METHODS

### Yeast strains and media

For the Yeast Metabolic Cycle, cells were continuously cultured in a minimal medium consisting of 5 g/l (NH_4_)_2_SO_4_, 2 g/l KH_2_PO_4_, 0.5 g/l MgSO_4_·7H_2_O, 0.1 g/l CaCl_2_·2H_2_O, 0.02 g/l FeSO_4_·7H_2_O, 0.01 g/l ZnSO_4_·7H_2_O, 0.005 g/l CuSO_4_·5H_2_O, 0.001 g/l MnCl_2_·4H_2_O, 1 g/l yeast extract, 10 g/l glucose, 0.5 ml/l 70% (vol/vol) H_2_SO_4_, and 0.5 ml/l Antifoam 204 (Sigma). For batch growth experiments, cells were grown in Synthetic Defined (SD) medium (Difco yeast nitrogen base and 2% glucose supplemented with amino acids [RDHILKMFTWYV], uracil, and adenine; or without supplementation for starvation experiments). The prototrophic *S. cerevisiae* strain CEN.PK was used for the YMC experiment. Unless otherwise stated, all other experiments utilized the strain BY4741, or derivatives thereof. Genomic knock-ins and knock-outs were generated using standard techniques (10). The *NOP2* coding sequence was replaced with the homologous sequence from *Kluyveromyces lactis* using the *URA3* pop-in/pop-out method, resulting in a marker-less strain with endogenous *NOP2* regulatory sequences. Plasmids carrying the promoter-gene hybrid reporters were generated using Gibson assembly (11), and the resulting expression cassettes were PCR-amplified and inserted at the dispensable *YHRCdelta14* LTR locus (12). Ribi transcription factors were C-terminally tagged with EGFP, and histone H2B was C-terminally tagged with mRuby2 (13). Strains, plasmids, and oligonucleotides used in this study are listed in Supplementary Tables S1–S3.

### Yeast metabolic cycle

A continuous culture of CEN.PK was established in a chemostat as previously described (14). Metabolic cycles were monitored using a dO_2_ probe, and 16 time points were chosen spanning a single cycle. At each time point, 20 ml of culture was mixed with 40 μl of a 50 mg/ml cycloheximide stock (in 100% ethanol), for a 100 μg/ml final concentration. The culture was shaken for 2 min, then centrifuged for 2 min at 4000 x g. The cell pellet was then re-suspended in ice-cold lysis buffer (20 mM Tris pH 8, 140 mM KCl, 5 mM MgCl_2_, 1 mM DTT, 100 μg/ml cycloheximide, 1% Triton X-100 and 0.025 U/μl Turbo DNase) and dripped into liquid nitrogen (ℓN_2_). Frozen droplets were stored at −80°C until further processing.

### Amino acid starvation

Cells were grown overnight at 30°C to saturation in SD, and then diluted to OD_600_ <0.1 in fresh SD medium. Cultures were incubated with vigorous shaking until the OD reached 0.4–0.6, and then half of the culture was centrifuged for 3 min at 3200 × g. The pellet was re-suspended in prewarmed SD medium lacking amino acids, and cells were shaken for an additional 20 min. CHX was then added at a final concentration of 100 μg/ml (for the matched replete sample, CHX was added to the remaining half of the culture that was not starved), and cultures were shaken for an additional 2 min before centrifuging for 3 min at 3200 × g. Pellets were re-suspended in ice-cold lysis buffer and frozen drop-wise in ℓN_2_ as described for the YMC.

For experiments without CHX pretreatment, 20 min-starved (or replete) cultures were transferred to a vacuum filtration apparatus and cells were collected on a 0.45 μm cellulose nitrate membrane (Whatman). Cells were then quickly scraped from the membrane with a metal spatula and immediately plunged into ℓN_2_. Frozen cells were stored at −80°C until further processing.

### Ribosome profiling and RNA-seq

Cells plus lysis buffer were cryogenically pulverized in a SPEX 6870 Freezer/Mill for 1 min at 15 cycles per min (for vacuum-filtered samples, frozen droplets of lysis buffer were added to the cells). The lysate powder was thawed and immediately clarified by two sequential centrifugation steps at 4°C: first for 5 min at 3000 × g, and then for 10 min at 20 000 × g.

Ribosome footprinting and library generation were carried out essentially as described in (15). RNA fragments from ∼26–34 nt were selected following RNase I digestion and PAGE separation. Barcode sequences were included on 3′ cloning linkers, and samples with unique barcodes were pooled together post-ligation when possible. A dual rRNA depletion strategy was employed, first with Ribo-Zero Gold for Yeast (Illumina), and then with biotinylated antisense oligos against rRNA species that co-migrate with ribosome footprints as described in (9).

For RNA-seq, RNA was first purified from clarified lysates using TRIzol (Invitrogen) and then rRNA was removed using Ribo-Zero Gold for Yeast. rRNA-depleted RNA was used to generate TruSeq Stranded libraries (Illumina) per the manufacturer's protocol. Ribosome profiling and RNA-seq libraries were sequenced on an Illumina HiSeq 4000 in single read 50-base mode. Each set of matched ribosome profiling and RNA-seq data is derived from a single biological sample.

### Sequencing data analysis

For ribosome profiling libraries generated in this study, linker sequences were removed from sequencing reads and samples were de-multiplexed using FASTX-clipper and -barcode splitter, respectively (http://hannonlab.cshl.edu/fastx_toolkit/). Unique molecular identifiers and sample barcodes were then removed from reads using a custom Python script. Bowtie v1.1.2 (http://bowtie-bio.sourceforge.net/) was used to filter out reads aligning to rRNAs and tRNAs, and all surviving reads were aligned to the *Saccharomyces cerevisiae* genome using tophat v2.1.1 (https://ccb.jhu.edu/software/tophat/). Counts per gene and normalized counts per gene (in reads per kilobase per million mapped reads, or RPKM) were calculated using the plastid cs program (16), with counts assigned to ribosomal P-sites determined by plastid's psite program. Regions of the yeast genome that could not be uniquely mapped from a 26-base read with two mismatches were identified using plastid's crossmap program, and these regions, along with the first 30 and last five codons of each coding sequence (CDS), were masked from RPKM calculations (for some analyses, cytoplasmic ribosomal protein abundances were separately quantified without crossmap masks, since most of these genes have nearly identical paralogs in *S. cerevisiae*). RNA-seq data were processed in the same manner, except reads first had to be reverse-complemented due to the TruSeq Stranded chemistry, and counts were assigned to the 5′-most aligned base. For viewing alignments, wiggle files were generated from genome alignments using plastid's make_wiggle program, and data were visualized in the IGV browser (http://software.broadinstitute.org/software/igv/).

For genes to be included in downstream analyses, they were required to have at least 128 mRNA counts (or 32 mRNA counts for the meiosis data from (9)) and at least 1 footprint count in the CDS. Genes listed as dubious ORFs in the Saccharomyces Genome Database (SGD, https://www.yeastgenome.org/) were not considered for analysis. Translation efficiencies were calculated for each gene by dividing the CDS footprint RPKM by the CDS mRNA RPKM, and fold changes in mRNA abundance and TE were normalized to set the median fold change of all genes to 1. Ribosome biogenesis factors were defined as non-ribosomal proteins annotated as ‘ribosomal small subunit biogenesis’ or ‘ribosomal large subunit biogenesis’ in the SGD Go Slim database. Data were analyzed and plotted using Pandas, Matplotlib, and Seaborn Python libraries.

### RT-qPCR

Amino acid starvation was carried out as described for ribosome profiling libraries, except cells were mock-treated with vehicle (ethanol or DMSO) in the no-CHX samples. Cells were harvested prior to adding CHX, and then 3, 6 and 9 min post-CHX by centrifuging 1 ml for 20 s at 11 000 × g. After aspirating the supernatant, cell pellets were immediately frozen in ℓN_2_, and RNA was purified by phenol-chloroform extraction and ethanol precipitation (17). Residual genomic DNA was degraded using a TURBO DNA-free kit (Ambion). Oligo(dT) primers were annealed to mRNA, and cDNA was generated using AMV reverse transcriptase (Promega). *NOP2* and *ACT1* (nucleolar protein 2, ribi gene; actin, housekeeping reference) cDNAs were quantified on a Roche LightCycler 480 instrument using GoTaq (Promega) PCR reactions containing SYBR Green (Life Technologies), and relative abundances were calculated using the 2^−ΔΔCt^ method (18). The starvation plus rapamycin experiment was conducted as described above, except cells were incubated for 20 min in starvation medium containing 200 nM rapamycin (Sigma) or vehicle (DMSO) prior to adding CHX. All qPCR experiments were conducted with technical triplicates.

### Time lapse fluorescence microscopy

Cells containing RFP-tagged histones and GFP-tagged ribi transcription factors were grown to OD_600_ 0.5–0.7 in SD medium and loaded into a CellASIC ONYX multi-chamber microfluidic plate for haploid yeast (Millipore). The growth chamber was perfused with SD medium at 10.8 kPa for 15 min, and then the media source was switched to SD lacking amino acids. Following 15 min of starvation the media was again switched to SD lacking amino acids plus 100 μg/ml CHX. Variations of the above scheme were also used to assess (i) the response to 200 nM rapamycin in the starvation medium, (ii) the response when CHX is not added after 20 min of starvation, and (iii) the baseline response to switching between replete media sources. For each strain/condition, two fields of view were imaged every 5 min on an inverted Nikon Ti microscope with a 40x objective (brightfield, GFP, and mCherry channels). The plate was maintained at 30°C during image acquisition. For rapamycin experiments without microfluidics, cells were transferred to glass-bottomed wells coated with Concanavalin A and allowed to settle for 5 min. Wells were then washed twice with the appropriate medium to remove non-adhered cells, and images were acquired as described above.

### Image analysis

The ratio of nuclear to cytoplasmic GFP-tagged transcription factors was determined using CellProfiler 3.1.5 (http://cellprofiler.org/). Nuclei were initially detected based on HTB2-mRuby2 signal, and then cell boundaries surrounding nuclei were defined based on GFP signal using the Propagation method with Robust Background thresholding. Cytoplasm was defined as the region of the cell not occupied by the nucleus. For each cell, the mean GFP intensities in the nucleus and cytoplasm were normalized to the mean GFP intensity of the entire cell. Nuclear localization was calculated as the normalized nuclear intensity divided by the normalized cytoplasmic intensity. A minimum of 200 cells were analyzed per strain/condition in the microfluidic experiments; 28–112 cells in glass-bottom wells.

## RESULTS

### Ribi transcripts are under apparent dynamic translational regulation during the YMC, meiosis, and amino acid starvation

**Figure 1.**
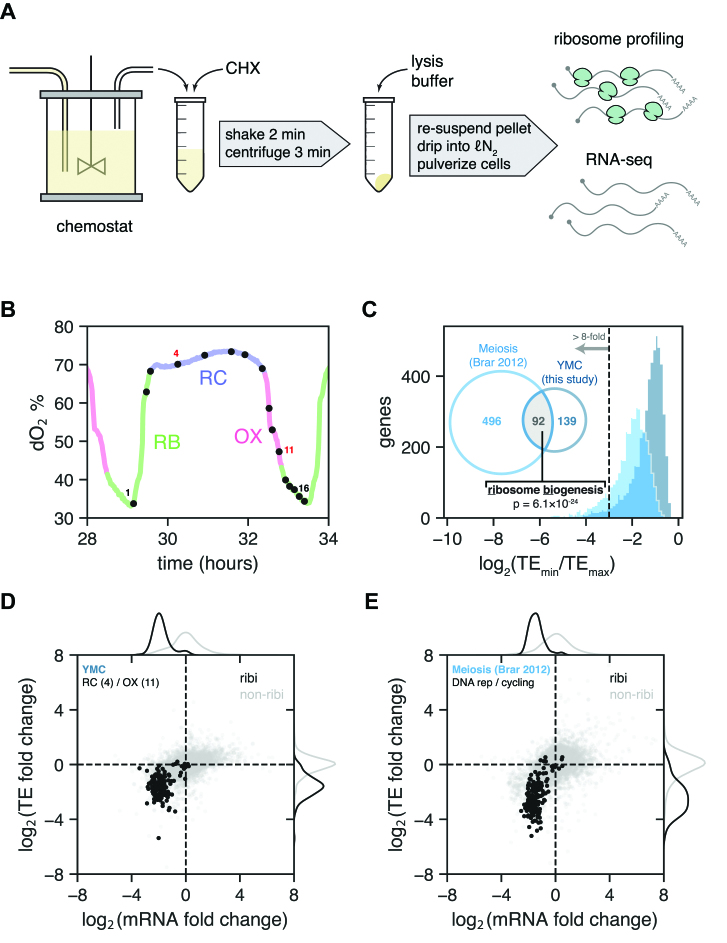
Apparent translational control of ribi genes in the Yeast Metabolic Cycle and meiosis. (**A**) Harvesting scheme from the YMC chemostat for ribosome profiling and RNA-seq. (**B**) Periodic fluctuations in dissolved oxygen for the span of the YMC during which samples were taken. Samples are numbered 1 through 16; samples 4 and 11 (red) were used to compare TE fold changes in panel D. RB, reductive building; RC, reductive charging; OX, oxidative phase. (**C**) Histograms of TE fold change (min/max) for all genes in the YMC and meiosis (9). Venn diagram shows the number of genes in each experiment exceeding 8-fold TE change, with the intersection highly enriched for ribosome biogenesis genes (SGD GO Process finder). (**D**) TE fold change vs. mRNA fold change for all ribi (black) and non-ribi (grey) genes between the RC and OX phases (YMC time points 4 and 11, respectively). Kernel density estimates of the distributions are plotted in the margins. (**E**) TE fold change vs. mRNA fold change, as in panel D, between the cycling vegetative and DNA replication time points of meiosis (9).

To obtain a global view of translational regulation during the different phases of the YMC, we performed ribosome profiling and RNA-seq at multiple YMC time points. Cells were grown in a chemostat, removed at defined time points, immediately treated with CHX to arrest translation, and subsequently processed to generate ribosome profiling and mRNA libraries suitable for analysis by next generation sequencing (Figure 1A). In total, 16 time points spanning a single ∼4.5-h cycle were analyzed (Figure 1B, Supplementary Figure S1A). At each time point, TEs were calculated for each protein-coding gene by dividing the gene's normalized ribosome profiling counts by its normalized RNA-seq counts (footprint RPKM/mRNA RPKM). We then quantified the range of TEs exhibited over the span of the YMC for each gene by dividing its minimum TE in the time course by its maximum TE. By this metric the median TE change for all genes during the YMC is 2.2-fold, with genes in the 99th percentile changing TE by at least 20-fold (Figure 1C, darker shade).

The same analysis was applied to previously published ribosome profiling and RNA-seq data acquired along a meiosis time course in yeast (9). In this context the median change in TE is 3.9-fold (Figure 1C, lighter shade); however, both distributions have long tails extending into the range of 100-fold or more. To investigate whether any of the strongly translationally regulated genes are shared between these distinct biological processes, we selected genes with TE change of 8-fold or greater from each experiment and analyzed the overlap of these gene sets. Remarkably, nearly half of the genes under strong translational regulation in the YMC are also strongly regulated in meiosis (Supplementary Figure S1B). Moreover, the overlapping gene set is highly enriched for ribi factors (*P* = 6 × 10^−24^) supporting the possibility of a common regulatory mechanism.

In addition to displaying a wide range of TEs in the YMC and meiotic time courses, the TEs of individual ribi genes are also highly correlated throughout each time course (Supplementary Figures S1C-D). By comparing TE and mRNA levels between high and low ribi gene translation states, a similar pattern emerges for both time courses in which decreased TE coincides with decreased mRNA content for the ribi genes (Figure 1D–E). Moreover, a previously published ribosome profiling study showed similar decreases in TEs and mRNA levels of ribi genes during amino acid starvation (8). Taken at face value, these observations suggest that a large group of genes involved in the energetically demanding ribi pathways are coordinately regulated in their mRNA abundances and translation efficiencies.

### Observed decrease in ribi TE during amino acid starvation is CHX-dependent

**Figure 2.**
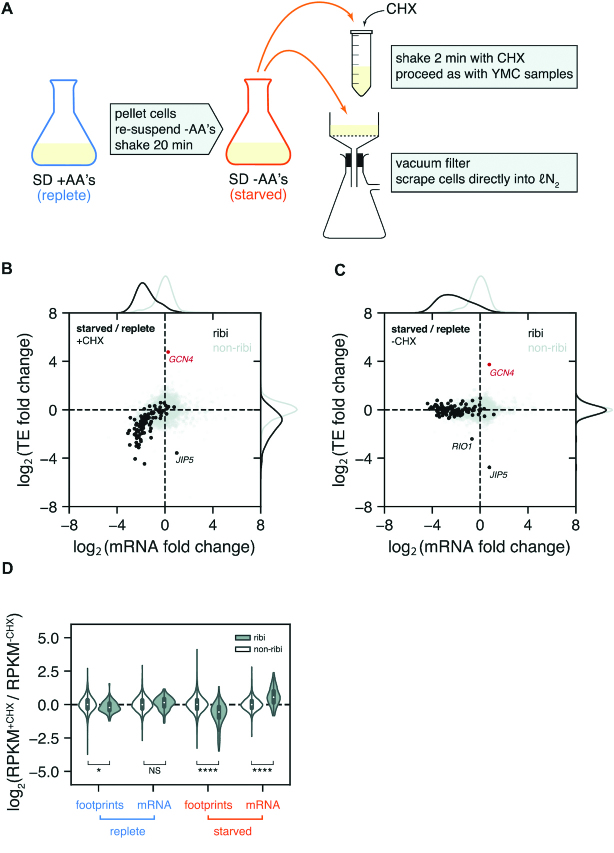
Effect of CHX on ribi gene TEs during amino acid starvation. (**A**) Amino acid starvation harvesting scheme with CHX (top) or without CHX (bottom). (**B**) TE fold change vs. mRNA fold change in starved vs. replete cells, with CHX pretreatment. As in the YMC and meiosis, ribi genes decrease along both dimensions. *GCN4* translational activation is a hallmark of eIF2α phosphorylation in starved cells. (**C**) Without CHX pretreatment, ribi TEs are unchanged except for *RIO1* and *JIP5*. (**D**) Violin plot of normalized count ratios with and without CHX pretreatment. The plot is separated by treatment (replete vs. starved), measured quantity (footprints vs. mRNA), and gene set (ribi vs. non-ribi). In starved cells, CHX pretreatment causes a decrease in footprints of ribi genes and an increase in mRNA levels of ribi genes, whereas non-ribi genes are relatively unaffected. This is also observed in replete cells but to a lesser degree. *P < 10^−2^, ****P < 10^−25^, NS not significant; two-sided *t*-test.

To understand the mechanistic basis of this effect we chose to focus on the amino acid starvation because, of the three transitions where we observed coordinated changes in the measured TE of ribi genes, this is the most experimentally facile. We were able to replicate the apparent change in TE during amino acid starvation using a slightly modified protocol in order to maintain consistency with the YMC harvesting scheme (Figure 2A, top). Cells were switched from minimal glucose medium with amino acids to minimal glucose medium without amino acids, and after 20 min of amino acid starvation we observed marked reductions in TEs and mRNA abundances of ribi genes as well as a pronounced increase in *GCN4* TE (Figure 2B), which is a hallmark of starvation-induced eIF2α phosphorylation.

Up to this point all of the experiments showing changes in ribi gene TEs were conducted with CHX pretreatment, which has been well-documented to impact ribosome profiling measurements by causing accumulation of ribosomes near translation start sites (8,19), and skewing the distribution of ribosome positions in a codon-dependent manner (20,21). However, since we took appropriate precautions such as masking footprint reads from the beginning of ORFs, such effects should have a minimal impact on the measure of the average ribosome density used to determine the overall rate of translation of a message; a key component of TE measurements. Nonetheless, we wanted to ensure that the TE measurements were not affected by CHX. We therefore used an alternative harvesting protocol that does not require CHX treatment, but instead relies on rapid filtration and freezing to arrest translation (Figure 2A, bottom). Ribosome profiling and RNA-seq were then carried out using the standard protocol.

Remarkably, we found that the change in ribi gene TEs was essentially abolished when cells were not treated with CHX (Figure 2C). Only two ribi genes, *JIP5* and *RIO1*, still exhibited large decreases in TE. *JIP5*'s proximity to an upstream gene makes it difficult to assess whether the transcript architecture changes in response to starvation; on the other hand, it is clear that the *RIO1* transcript is significantly extended on the 5′ end in starved cells, which incorporates a uORF that appears to sequester ribosomes away from the canonical ORF (Supplementary Figure S2A). This ‘long undecoded transcript isoform’, or LUTI, represents a pervasive regulatory mechanism that was recently described in meiosis (22–24).

In order to determine how CHX pretreatment might lead to an apparent low TE for the ribi genes, we first compared normalized counts per gene in starvation experiments with and without CHX (Figure 2D, Supplementary Figure S2B). For the vast majority of non-ribi genes, CHX treatment did not have a substantial effect on measured gene expression in any condition. However, the ribi genes experienced significant CHX-dependent changes in both footprint and mRNA abundances, and this effect was greatly exaggerated in starved cells. Somewhat paradoxically, pretreatment with CHX leads to increased mRNA and decreased footprint counts for ribi transcripts, which conspire to make TE measurements much lower in starvation experiments that include a CHX pretreatment step.

### CHX causes rapid induction of ribi transcripts in starved cells

**Figure 3.**
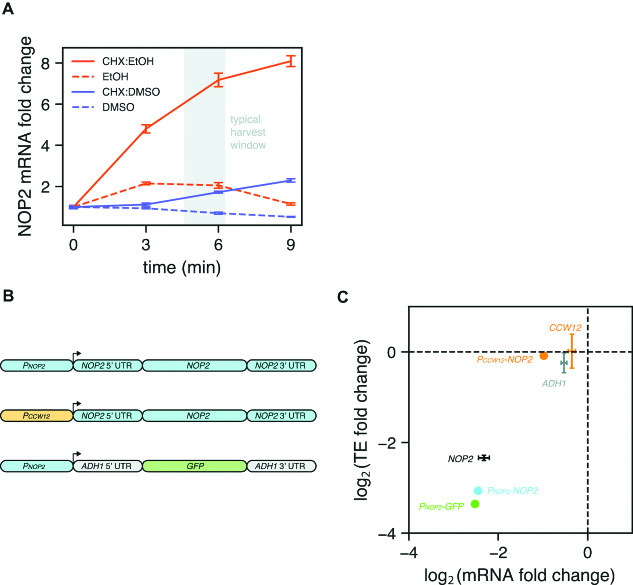
CHX influences the TE of a ribi gene via transcription. (**A**) CHX or vehicle was added to starved cells at *t* = 0 and relative abundance of a ribi mRNA (*NOP2*) was monitored over time. *NOP2* transcripts accumulate faster and to a greater degree when CHX is dissolved in ethanol as opposed to DMSO. The shaded area indicates the typical amount of elapsed time between CHX pretreatment and freezing of cells for ribosome profiling experiments in this study. Error bars represent the standard deviation of three technical replicates from a single biological sample. (**B**) Reporter constructs with various promoter, coding sequence, and UTR elements were integrated into the dispensable *YHRCdelta14* locus. Sequences from the ribi gene *NOP2* are shaded blue. (**C**) TE fold change versus mRNA fold change for reporter strains in panel B following amino acid starvation. TE and mRNA only decrease when the *NOP2* promoter is driving expression. Fold changes for endogenous genes (*NOP2, CCW12, ADH1*) are represented as mean ± standard deviation across 2–4 independent experiments.

A simple explanation for the difference in mRNA abundances with and without CHX pretreatment would be that CHX induces transcription of ribi genes. To test this possibility, we treated starved cultures with CHX or vehicle and measured the mRNA abundance of a typical ribi gene, *NOP2*, using RT-qPCR over a 9-min time course. Surprisingly, *NOP2* mRNA abundance increased in both the CHX- and vehicle-treated samples, although the magnitude of the increase was much larger with CHX (Figure 3A, orange lines). In this case the vehicle was ethanol, which is the drug manufacturer's recommended solvent. The working solution was made at a 500× concentration, therefore the quantity of added ethanol in the culture after treatment was 0.2% by volume. We next tested whether the response could be replicated with larger or smaller amounts of ethanol alone. Indeed, treatments of 0.4% and 0.1% ethanol proportionally scaled the *NOP2* mRNA increase relative to 0.2% ethanol (Supplementary Figure S3A).

Since CHX is also routinely dissolved in dimethyl sulfoxide (DMSO), we repeated the same experiment with CHX in DMSO or with DMSO alone. While *NOP2* mRNA abundance did increase following several minutes of drug treatment, the magnitude of the increase was 3- to 4-fold smaller compared to treatment with CHX in ethanol (Figure 3A, purple lines). This discrepancy does not appear to be due to gross differences in the activity of CHX in each solvent, since both formulations arrested growth with equal potency (Supplementary Figure S3B). Interestingly, the response to ethanol is strain-specific, as a prototrophic strain treated with CHX in ethanol exhibits a similar *NOP2* mRNA increase as our auxotrophic lab strain treated with CHX in DMSO (Supplementary Figure S3C). Therefore, in yeast starvation experiments that include CHX pretreatment—especially if the strain is auxotrophic and CHX is dissolved in ethanol—cells accumulate ribi transcripts from the time CHX is added to the culture until cells are frozen in liquid nitrogen.

We reasoned that if the apparent TE decrease in starved cells is due to transcription of new ribi messages following CHX treatment, then a ribi promoter should be necessary and sufficient for the effect. Since most ribi genes are essential in yeast, we started by replacing the endogenous *NOP2* coding sequence with a homologous sequence from *K. lactis* which contains enough nucleotide differences to be readily distinguished from the *S. cerevisiae* sequence using deep sequencing. We then introduced different *NOP2* reporter constructs elsewhere in the genome, allowing us to unambiguously quantify their expression via ribosome profiling and RNA-seq, and thus determine the influence of promoters, UTRs, and coding sequences on TE (Figure 3B, Supplementary Figure S3D). As expected, with CHX pretreatment, a fully wild-type *NOP2* gene exhibited decreases in TE and mRNA levels following amino acid starvation (Figure 3C). However, when *NOP2* was instead transcribed from the non-ribi *CCW12* promoter, the decrease in mRNA abundance was severely attenuated and the change in TE was eliminated entirely. Finally, an exogenous GFP sequence flanked by non-ribi *ADH1* UTRs transcribed from the *NOP2* promoter exhibited decreases in mRNA and TE similar to those observed for wild-type *NOP2*. Collectively, these reporter experiments demonstrate that a ribi promoter is both necessary and sufficient to recapitulate the low TEs observed in amino acid-starved cells when CHX pretreatment is used.

### CHX-induced ribi transcription requires TORC1 signaling

**Figure 4.**
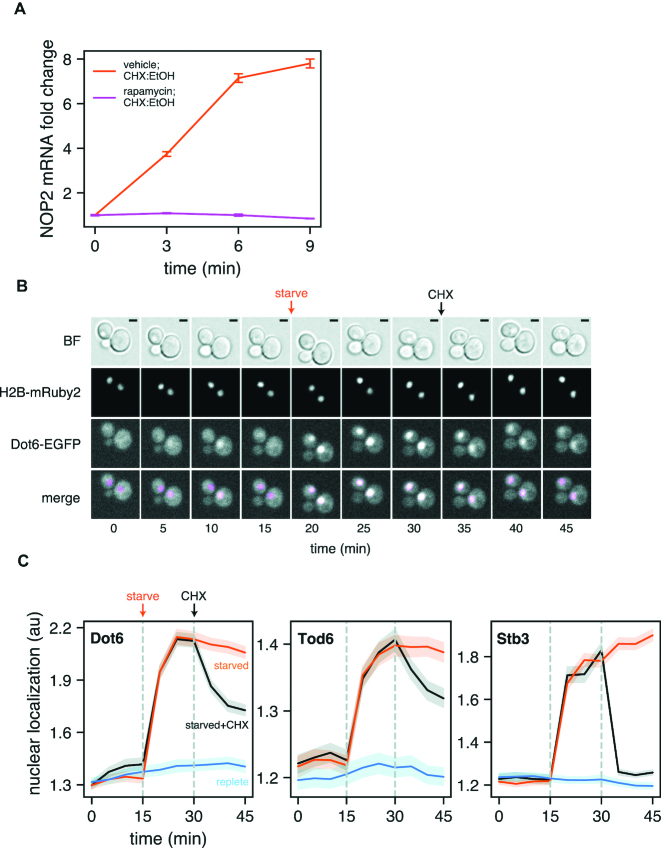
CHX-induced ribi gene transcription requires TORC1 signaling. (**A**) Cells were transferred to starvation medium and simultaneously treated with rapamycin or vehicle (DMSO). After 20 min CHX was added and *NOP2* mRNA abundance was monitored over time. Rapamycin treatment abolishes transcriptional activation. Error bars represent the standard deviation of three technical replicates from a single biological sample. (**B**) Dot6 and histone H2B were tagged with EGFP and mRuby2, respectively, and cells were immobilized in a CellASIC microfluidic device. Images were acquired as replete medium was flowed over the cells for 15 min, followed by 15 min of starvation medium, and finally 15 min of starvation medium plus CHX. Dot6 rapidly localizes to the nucleus upon starvation and exits the nucleus after CHX treatment. Nuclei are false-colored magenta in the merged image. BF, brightfield with 2 μm scale bar. (**C**) The experiment in panel B was conducted with EGFP-tagged Dot6, Tod6, and Stb3; cells were also kept in replete medium (blue) or not treated with CHX (orange) as controls. Nuclear localization was defined as the fraction of GFP signal in the nucleus relative to the signal in the cytoplasm. All three transcription factors exit the nucleus upon CHX treatment; Stb3 responds most robustly. Localization data is plotted as the mean of all cells observed (*n* = 213–310) with 95% confidence interval.

We next sought to identify the factors mediating the transcriptional response of ribi genes to CHX. The Target Of Rapamycin (TOR) protein kinase emerged as a candidate given its central role in regulating ribosome biogenesis in response to nutrient availability (25). In budding yeast TOR is a member of two protein complexes, TORC1 and TORC2, the former of which promotes growth when conditions are favorable, and is inactivated by the macrolide antibiotic rapamycin (26). Upon nutrient starvation or rapamycin treatment, reduced TORC1 signaling leads to inhibition of rRNA and ribosomal protein gene transcription, as well as global attenuation of translation initiation (27). Since ribi gene transcription was reactivated in the presence of CHX despite poor nutrient conditions, we speculated that the TORC1 pathway might be involved. To test this possibility, we measured changes in *NOP2* mRNA abundance induced by CHX in the presence and absence of rapamycin. After shifting cells to starvation medium containing rapamycin or vehicle and incubating for 20 minutes, CHX was added and *NOP2* mRNA abundance was monitored over time by qPCR. *NOP2* transcript levels increased by nearly 8-fold in vehicle-treated cells but remained unchanged in rapamycin-treated cells (Figure 4A), demonstrating that TORC1 signaling is required for CHX-mediated ribi transcription.

Expression of ribi genes is specifically regulated by at least three transcription factors—Dot6, Tod6, and Stb3—which bind two conserved sequence motifs in ribi promoters to repress transcription (28). These factors are phosphorylated by the kinase Sch9, itself a direct target of TORC1 (29), which is thought to mediate their nuclear localization and/or chromatin binding affinity (30). To assess whether CHX treatment affects the sub-cellular localization of these factors, we C-terminally tagged each with EGFP and monitored their localization in live cells. Using a microfluidic device, we were able to rapidly switch between different media and image the same cells over time. In replete medium, all three transcription factors are evenly distributed throughout cells, and within 5 min of amino acid starvation they accumulate in the nucleus. Strikingly, within 5 min of treating starved cells with CHX, all three transcription factors begin to exit the nucleus (Figure 4B–C), and after 15 min nuclear localization is reversed by 35–50% (Tod6, Dot6) or up to 90% (Stb3). Rapamycin did not prevent these factors from leaving the nucleus upon CHX exposure using this experimental setup (Supplementary Figure S4A), however it is well-documented that small molecules can be absorbed by the polymer that constitutes the channels of this microfluidic device (31–33). After repeating the rapamycin/CHX treatment regimen in a flask and imaging in glass-bottom wells, we observed significantly more nuclear localization post-CHX for all three transcription factors in rapamycin-treated cells (Supplementary Figure S4B). Taken together, these data suggest that CHX works through TORC1 and Sch9 to relieve transcriptional repression of ribi genes, despite poor nutrient availability.

## DISCUSSION

Ribosome profiling provides an unprecedented view into the absolute rate of translation, and paired with mRNA measurements, translation efficiency. However, such measurements depend on the ability to accurately freeze the translational state of a cell which can be challenging due to the rapid kinetics of translation initiation and elongation. To overcome this challenge, translation elongation inhibitors such as CHX are often used to ‘lock’ ribosomes in place prior to harvesting. Such treatment comes with well-described caveats such as accumulation of ribosome density near translation start sites, and ‘smearing’ of ribosome density in gene bodies. However, with proper precautions, these effects typically have minimal impact on the overall rate of translation as measured by the average ribosome density across the body of an mRNA. The present work adds the prospect of rapid CHX-induced transcription as a distinct and potentially more pervasive artifact when one is attempting to measure TE.

Here, we show that what appears to be strong translational regulation of a large group of ribi genes during amino acid starvation is in fact an experimental artifact caused by CHX pretreatment. Even brief drug exposure (5–6 min) leads to a substantial increase in ribi mRNAs, and due to the presence of CHX, these new messages cannot be translated. This together with a decrease in ribosome-protected footprints of ribi genes leads to artificially low measured translation efficiencies. Using *NOP2* as a representative ribi gene, we find that the increased ribi mRNA content in CHX-treated samples is due to rapid accumulation of transcripts upon drug exposure. Remarkably, the solvent used to make a CHX working solution has a large effect on the response to the drug: while CHX dissolved in DMSO leads to a slight increase in mRNA after 9 min, CHX dissolved in ethanol multiplies the increase by as much as 4-fold. Even more surprising is the observation that as little as 0.1% ethanol by itself is enough to transiently increase *NOP2* levels, but only in an auxotrophic lab strain. This heightened sensitivity to ethanol could be the result of abnormal utilization of glucose by auxotrophs, which may respond more readily to otherwise unfavorable carbon sources such as ethanol and increase expression of pro-growth genes (34). Since the *NOP2* increase from CHX in ethanol is much greater than the sum of the individual increases induced by CHX in DMSO or ethanol alone, CHX and ethanol may influence ribi mRNA abundance through separate pathways.

The CHX-dependent decrease in ribi gene ribosome-protected footprints is surprising in light of prior observations that CHX inhibits mRNA decay (35), therefore one might assume *a priori* that CHX treatment would actually increase relative footprint abundances in cases when transcription is shut off (e.g. ribi genes during starvation) and old mRNAs are turned over. Notably, ribi transcripts are highly enriched in certain codons for aspartate, glutamate, and lysine (Supplementary Figure S2C), and all genes enriched in these codons—ribi or otherwise—tend to have fewer footprint counts in CHX-treated samples (Supplementary Figure S2D). Although this effect of CHX on footprint counts remains enigmatic, it is possible that when charged tRNA pools are depleted (as is the case during amino acid starvation), in the presence of CHX, ribosomes engaged in translating these codons are more likely to encounter collisions that trigger ribosome quality control pathways, resulting in clearance of these ribosomes from the mRNA (36).

**Figure 5.**
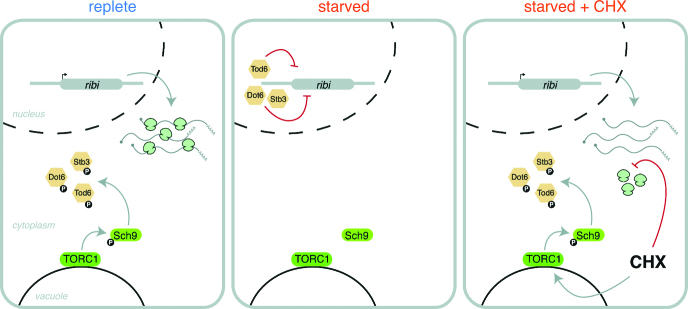
Proposed model of the influence of CHX on ribi gene TEs. In replete conditions TORC1 signaling is active, leading to inhibitory phosphorylation of ribi gene transcriptional repressors and active transcription/translation of ribi genes. In starved cells, TORC1 signaling decreases, thereby allowing ribi transcriptional repression. Upon adding CHX to starved cells, TORC1 signaling resumes and new ribi transcripts are produced. In the presence of CHX, however, these mRNAs cannot be translated and apparent TE is low.

When conditions are unfavorable for growth, ribi gene expression is shut off by at least three transcriptional repressors. Using time-lapse fluorescent microscopy, we show that adding CHX to starved cells causes these factors to exit the nucleus within 5 min. These factors are phosphorylated by the TORC1 substrate Sch9 to regulate their activity, and Sch9 phosphorylation is known to increase in response to CHX (29). Our observations are consistent with a model in which CHX activates TORC1 signaling in starved cells, overriding the starvation response and causing nuclear export of the ribi gene transcriptional repressors. Transcription of the ribi genes thus resumes, but in the presence of CHX the new mRNAs are not translated, leading to what appears to be strong translational repression (Figure 5). Although we cannot formally rule out a CHX-driven change in ribi mRNA stability, the most parsimonious explanation given our observations is that the accumulation of mRNAs is due to increased transcription. In support of this hypothesis, we show that when CHX pretreatment is used to harvest cells, a ribi promoter sequence is necessary and sufficient to impart ‘low’ TE on a reporter gene following amino acid starvation, independent of UTR sequences. We also show that CHX causes rapid re-partitioning of ribi transcription factors within the cell, but blocking TORC1 signaling with rapamycin prior to adding CHX suppresses ribi transcriptional repressor egress from the nucleus and abolishes accumulation of a ribi transcript in starved cells. It is worth noting that ribi mRNA abundances still decrease following amino acid starvation in a strain with all three transcriptional repressor genes deleted, so additional factors are likely involved (Supplementary Figure S4C). It is also important to note that genes encoding ribosomal protein (RP) subunits are not subject to these effects, as their overall TE fold changes in the YMC and meiosis are comparatively low (Supplementary Figure S1B), and CHX pretreatment did not lead to increases in their measured mRNA abundances or decreases in measured ribosome footprints (data not shown). Interestingly, studies in the fission yeast *Schizosaccharomyces pombe* have shown CHX-dependent changes in RP gene TEs during nitrogen starvation and histidine biosynthesis inhibition, suggesting a similar CHX-induced mechanism operating on a distinct regulon (37,38). Ribi and RP genes are regulated by different sets of transcription factors in *S. cerevisiae* (39–41), and ribi gene expression peaks just before that of the RPs during the YMC, supporting the idea that decoupling ribi from RP gene regulation allows ribosome assembly machinery to accumulate before subunits are produced (42).

Although the precise mechanism of CHX-induced Sch9 phosphorylation remains unknown, a plausible explanation is that an increase in cytoplasmic concentrations of free amino acids following global translation arrest mimics a shift to nutrient-rich conditions, leading to TORC1 activation (43,44). Therefore, it is possible that other stress conditions resulting in Sch9 dephosphorylation could be subject to translation inhibitor artifacts. These conditions include carbon/nitrogen/phosphate starvation, osmotic stress, redox stress, and heat shock (29); and potentially affected genes in the TOR regulon are numerous. Indeed, cells in two distinct growth conditions—the YMC and meiosis, both involving carbon and/or nitrogen limitation—had similarly low TE of ribi genes when CHX pretreatment was used in the ribosome profiling protocol. CHX is an invaluable tool, but it is important to be aware of the complex interplay between drug formulation, culture conditions, and cell genotype that can lead to unexpected results. We hope that this work will aid future ribosome profiling experimental design by highlighting additional pitfalls one might encounter as a result of treating cells with CHX, as well as ways to mitigate them.

## DATA AVAILABILITY

The raw and processed sequencing data from this study have been submitted to the NCBI Gene Expression Omnibus under accession number GSE125038.

## Supplementary Material

gkz205_Supplemental_Files
