# Supplementary material for: Cycloheximide can distort measurements of mRNA levels and translation efficiency
Source: Nucleic Acids Res. 2019 Mar 27;47(10):4974–85. doi: 10.1093/nar/gkz205 (PMC6547433; doi:10.1093/nar/gkz205)
Supplement: gkz205_Supplemental_Files [file gkz205_supplemental_files.pdf]

Figure S1

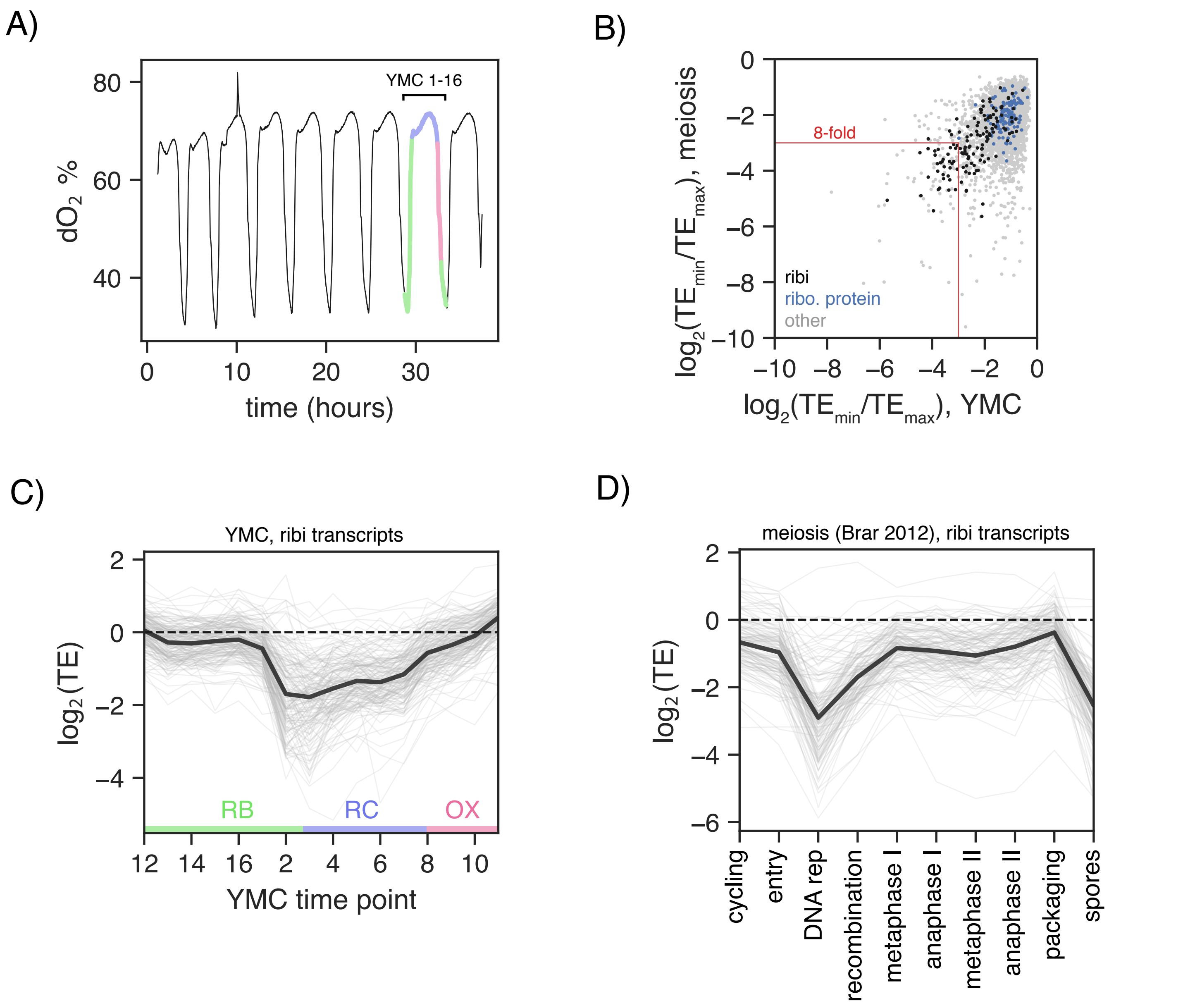
A)
YMC 1-16
dO2 %
time (hours)
B)
8-fold
ribi
ribo. protein
other
log2(TEmin/TEmax), meiosis
log2(TEmin/TEmax), YMC
C)
YMC, ribi transcripts
log2(TE)
RB
RC
OX
YMC time point
D)
meiosis (Brar 2012), ribi transcripts
log2(TE)
cycling
entry
DNA rep
recombination
metaphase I
anaphase I
metaphase II
anaphase II
packaging
spores

Figure S2

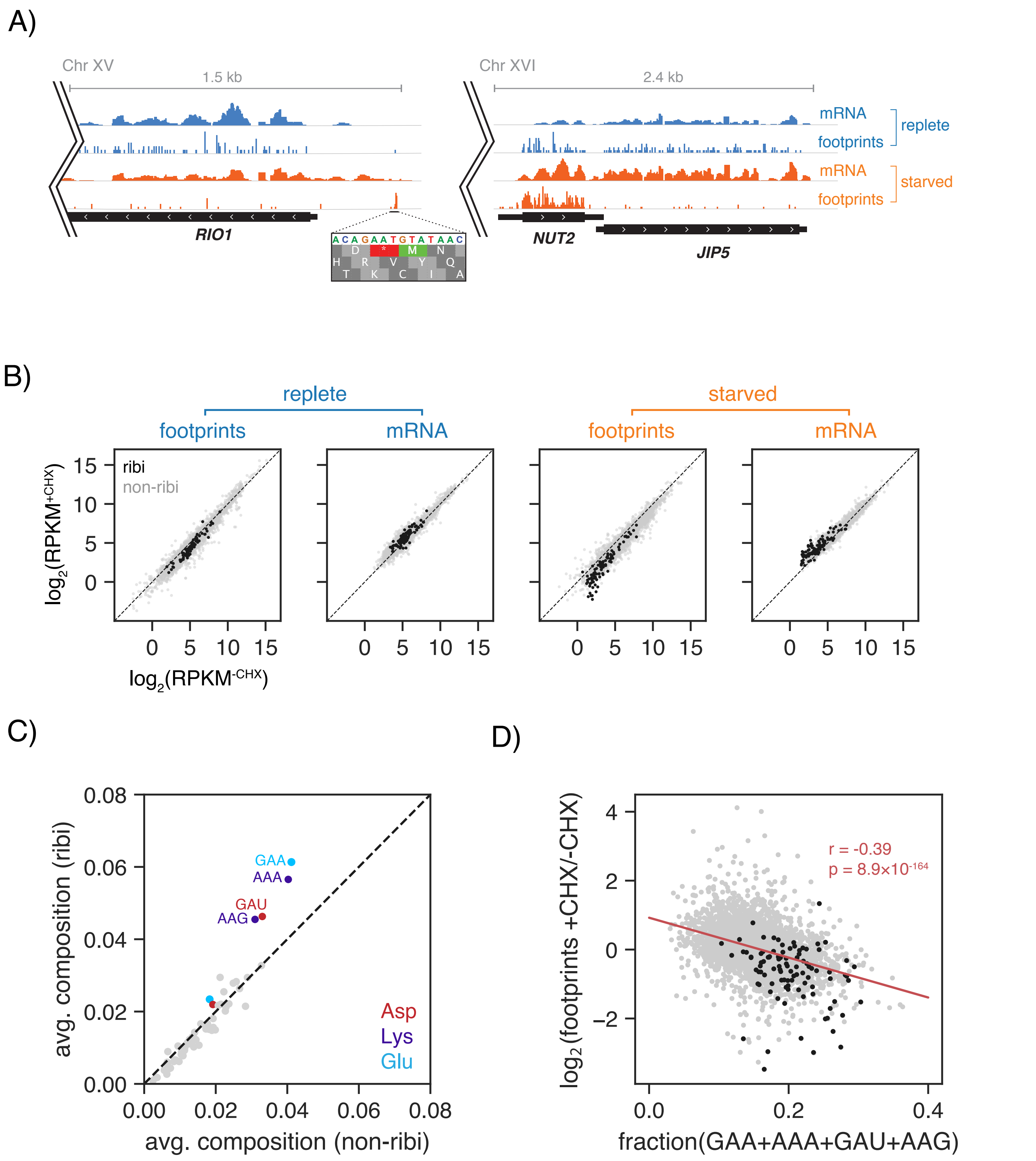
A)
Chr XV
1.5 kb
RIO1
ACAGAATGTATAAC
Chr XVI
2.4 kb
NUT2
JIP5
mRNA
footprints
replete
mRNA
footprints
starved
B)
replete
starved
footprints
mRNA
footprints
mRNA
ribi
non-ribi
log2(RPKM+CHX)
log2(RPKM-CHX)
C)
GAA
AAA
GAU
AAG
Asp
Lys
Glu
avg. composition (ribi)
avg. composition (non-ribi)
D)
r = -0.39
p = 8.9×10-164
log2(footprints +CHX/-CHX)
fraction(GAA+AAA+GAU+AAG)

Figure S3

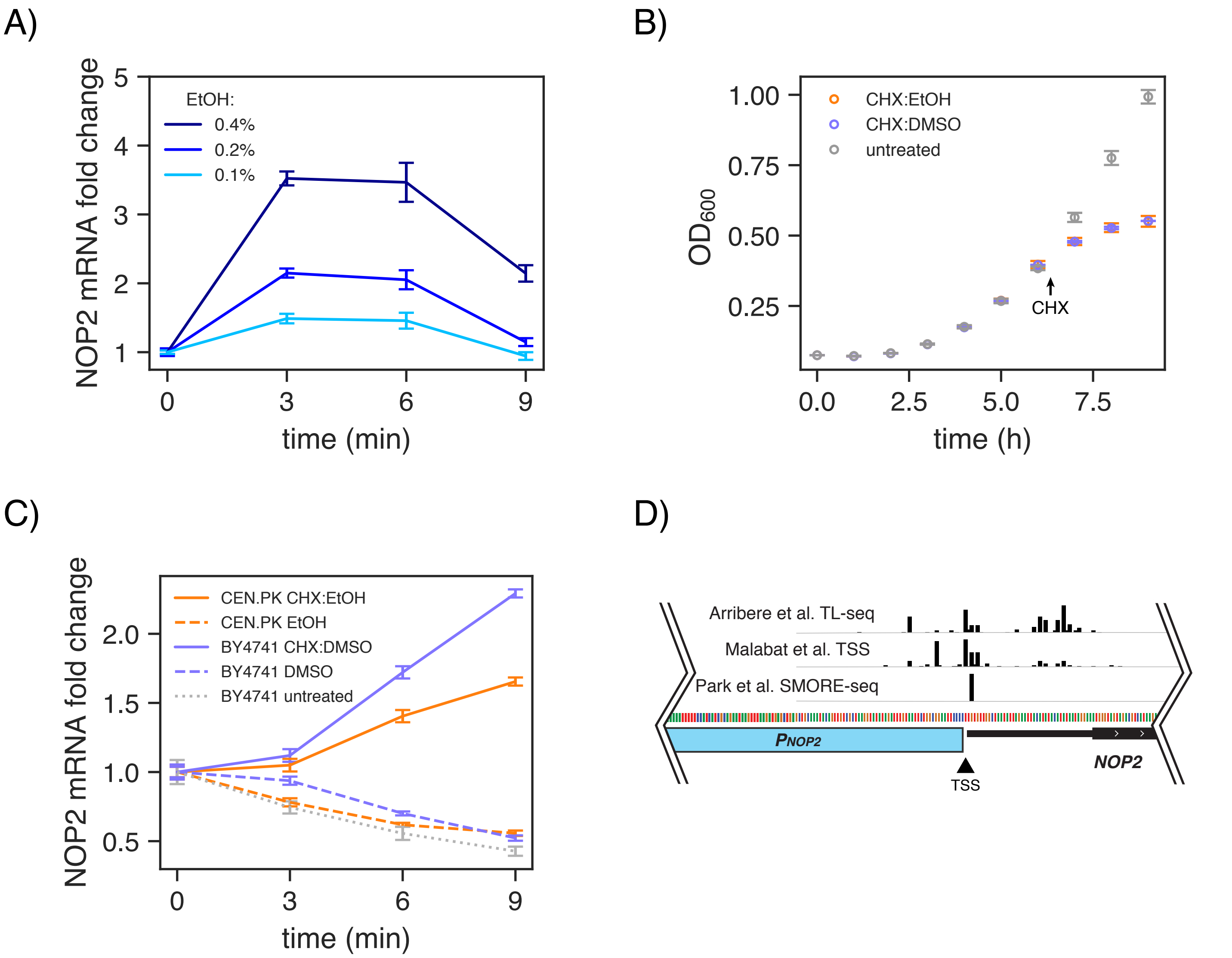
A)
EtOH:
0.4%
0.2%
0.1%
NOP2 mRNA fold change
time (min)
B)
CHX:EtOH
CHX:DMSO
untreated
OD_{600}
CHX
time (h)
C)
CEN.PK CHX:EtOH
CEN.PK EtOH
BY4741 CHX:DMSO
BY4741 DMSO
BY4741 untreated
NOP2 mRNA fold change
time (min)
D)
Arribere et al. TL-seq
Malabat et al. TSS
Park et al. SMORE-seq
PNOP2
TSS
NOP2

Figure S4

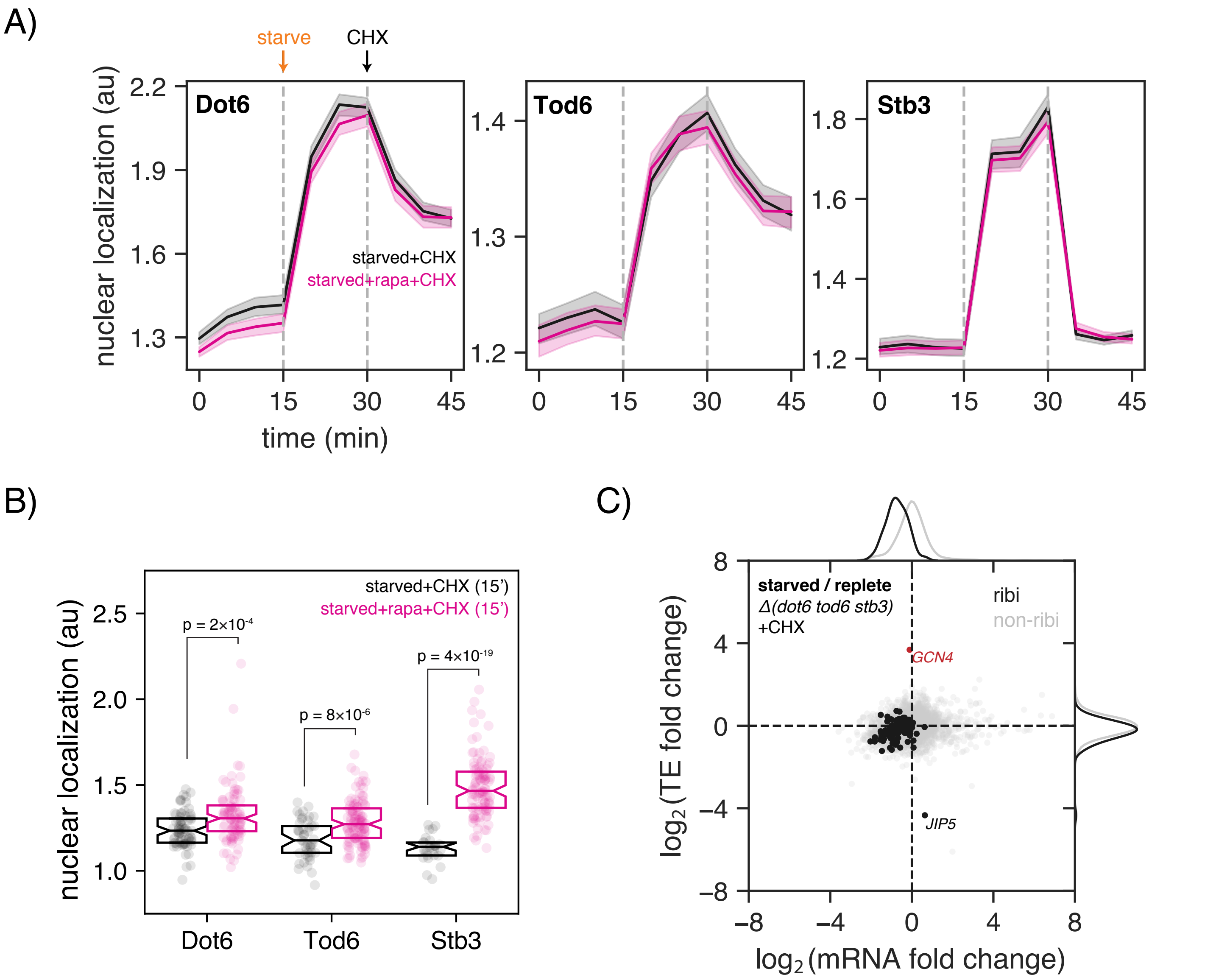
A)
starve
CHX
Dot6
Tod6
Stb3
nuclear localization (au)
time (min)
starved+CHX
starved+rapa+CHX
B)
starved+CHX (15')
starved+rapa+CHX (15')
p = 2×10-4
p = 8×10-6
p = 4×10-19
Dot6
Tod6
Stb3
C)
starved / replete
Δ(dot6 tod6 stb3)
+CHX
ribi
non-ribi
GCN4
JIP5
log2(TE fold change)
log2(mRNA fold change)

Supplementary Table S1. Strains used in this study

NAME	DESCRIPTION	MAT	GENOTYPE	PARENT	SOURCE
yJW1201	BY4741	a	*his3-Δ1 leu2-Δ0 met15-Δ0 ura3-Δ0*	S288C	Brachmann et al. (1998). PMID: 9483801
yJW1857	CEN.PKa	a	WT	sporulated from CEN.PK122	van Dijken et al. (2000). PMID: 10862876
yJW1860	*K.lac NOP2*	a	*nop2::klNOP2 his3-Δ1 leu2-Δ0 met15-Δ0 ura3-Δ0*	BY4741	this study
yJW1861	*K.lac NOP2;* P_{CCW12}*-NOP2*	a	*nop2::klNOP2 yhrcdelta14::*P_{CCW12}*-NOP2::kanMX6 his3-Δ1 leu2-Δ0 met15-Δ0 ura3-Δ0*	yJW1860	this study
yJW1862	*K.lac NOP2;* P_{NOP2}*-NOP2*	a	*nop2::klNOP2 yhrcdelta14::*P_{NOP2}*-NOP2::kanMX6 his3-Δ1 leu2-Δ0 met15-Δ0 ura3-Δ0*	yJW1860	this study
yJW1863	P_{NOP2}*-5'*UTR_{ADH1}*-EGFP-3'*UTR_{ADH1}	a	*yhrcdelta14::*P_{NOP2}*-5'*UTR_{ADH1}*-EGFP-3'*UTR_{ADH1}*::kanMX6 his3-Δ1 leu2-Δ0 met15-Δ0 ura3-Δ0*	BY4741	this study
yJW1864	*Δ(dot6 tod6 stb3)*	a	*dot6::LEU2 tod6::URA3 stb3::kanMX6 his3-Δ1 leu2-Δ0 met15-Δ0 ura3-Δ0*	BY4741	this study
yJW1865	Dot6-EGFP; H2B-mRuby2	a	*dot6::DOT6-EGFP::kanMX6 htb2::HTB2-mRuby2::SpHIS5 his3-Δ1 leu2-Δ0 met15-Δ0 ura3-Δ0*	BY4741	this study
yJW1866	Tod6-EGFP; H2B-mRuby2	a	*tod6::TOD6-EGFP::kanMX6 htb2::HTB2-mRuby2::SpHIS5 his3-Δ1 leu2-Δ0 met15-Δ0 ura3-Δ0*	BY4741	this study
yJW1867	Stb3-EGFP; H2B-mRuby2	a	*stb3::STB3-EGFP::kanMX6 htb2::HTB2-mRuby2::SpHIS5 his3-Δ1 leu2-Δ0 met15-Δ0 ura3-Δ0*	BY4741	this study

Supplementary Table S2. Plasmids used in this study

NAME	DESCRIPTION	PURPOSE	SOURCE
pFA6a-link-yoEGFP-Kan	EGFP-Kan	Tag ribi transcription factors with EGFP	Addgene 44900
pFA6a-link-yomRuby2-SpHis5	mRuby2-His	Tag H2B with mRuby2	Addgene 44858
pJW1746	pRS306_YHRCdelta14UP-P_{CCW12}-NOP2-KanR-YHRCdelta14DOWN	PCR template to generate yJW1861	this study
pJW1747	pRS306_YHRCdelta14UP-P_{NOP2}-NOP2-KanR-YHRCdelta14DOWN	PCR template to generate yJW1862	this study
pJW1748	pRS306_YHRCdelta14UP-P_{NOP2}-5'UTR_{ADH1}-EGFP-3'UTR_{ADH1}-KanR-YHRCdelta14DOWN	PCR template to generate yJW1863	this study

Supplementary Table S3. Oligonucleotides used in this study

NAME	SEQUENCE	PURPOSE	DESCRIPTION
oAF66	/5rApp/NNNNNATCGAGATCGGAAGAGCACACGTCTGAACTC/3ddC/	ribosome profiling	3' cloning linker (ATCG)
oAF67	/5rApp/NNNNNTAGCAGATCGGAAGAGCACACGTCTGAACTC/3ddC/	ribosome profiling	3' cloning linker (TAGC)
oAF68	/5rApp/NNNNNCGATAGATCGGAAGAGCACACGTCTGAACTC/3ddC/	ribosome profiling	3' cloning linker (CGAT)
oAF69	/5rApp/NNNNNGCTAAGATCGGAAGAGCACACGTCTGAACTC/3ddC/	ribosome profiling	3' cloning linker (GCTA)
oAF70	/5rApp/NNNNNAGTCAGATCGGAAGAGCACACGTCTGAACTC/3ddC/	ribosome profiling	3' cloning linker (AGTC)
oAF71	/5rApp/NNNNNGACTAGATCGGAAGAGCACACGTCTGAACTC/3ddC/	ribosome profiling	3' cloning linker (GACT)
oAF72	/5rApp/NNNNNCTGAAGATCGGAAGAGCACACGTCTGAACTC/3ddC/	ribosome profiling	3' cloning linker (CTGA)
oAF73	/5rApp/NNNNNTCAGAGATCGGAAGAGCACACGTCTGAACTC/3ddC/	ribosome profiling	3' cloning linker (TCAG)
oAF74	/5Phos/AGATCGGAAGAGCGTCGTGTAGGGAAAGAG/iSp18/CTGGAGTTCAGACGTGTG	ribosome profiling	RT
oAF75	AATGATACGGCGACCACCGAGATCTACACTCTTTCCCTACACGACGCTC	ribosome profiling	PCR (universal)
oAF76	CAAGCAGAAGACGGCATACGAGATTACAAGGTGACTGGAGTTCAGACGTGTGCTC	ribosome profiling	PCR (index 12)
oAF77	CAAGCAGAAGACGGCATACGAGATATTGGCGTGACTGGAGTTCAGACGTGTGCTC	ribosome profiling	PCR (index 6)
oAF78	CAAGCAGAAGACGGCATACGAGATGGAACTGTGACTGGAGTTCAGACGTGTGCTC	ribosome profiling	PCR (index 14)
oAF79	CAAGCAGAAGACGGCATACGAGATAAGCTAGTGACTGGAGTTCAGACGTGTGCTC	ribosome profiling	PCR (index 10)
oAF80	CAAGCAGAAGACGGCATACGAGATGCCTAAGTGACTGGAGTTCAGACGTGTGCTC	ribosome profiling	PCR (index 3)
oAF81	CAAGCAGAAGACGGCATACGAGATCGTGATGTGACTGGAGTTCAGACGTGTGCTC	ribosome profiling	PCR (index 1)
oAF82	CAAGCAGAAGACGGCATACGAGATCCACTCGTGACTGGAGTTCAGACGTGTGCTC	ribosome profiling	PCR (index 23)
oAF83	CAAGCAGAAGACGGCATACGAGATTTGACTGTGACTGGAGTTCAGACGTGTGCTC	ribosome profiling	PCR (index 13)
oDAS220	CGTCTGGATTGGTGGTTCTATC	qPCR	ACT1 (For)
oDAS221	GGACCACTTTCGTCGTATTCTT	qPCR	ACT1 (Rev)
oDAS880	TCTATCCGCCATTGATTCTGTT	qPCR	NOP2 (For)
oDAS881	CTATGACAGCTTCGTCCTCTTC	qPCR	NOP2 (Rev)

SUPPLEMENTARY FIGURE AND TABLE LEGENDS

Supplementary Figure S1.

(A) Overview of metabolic cycles observed over ~40 hours. Decreases in dissolved oxygen correspond to the OX phase of the YMC. Time points 1-16 are highlighted; detail shown in Figure 1B. (B) Scatter plot of the TE range (min/max) exhibited by each gene in meiosis vs the YMC. The set of genes with 8-fold or more TE change in both time courses (red box) is highly enriched for ribi factors, but not protein subunits of the cytoplasmic ribosome. (C) TEs of ribi genes over the YMC time course. Each gene is shown as a thin grey line; the median of all ribi genes is shown as a thick black line. In general, TEs appear to be low during the RB to RC transition, and high at the end of OX. (D) TEs of ribi genes through meiosis, from (1). TEs appear to be low during DNA replication and in spores.

Supplementary Figure S2.

(A) mRNA and footprint counts mapped to the *RIO1* and *JIP5* loci. In starved cells the *RIO1* transcript is extended on the 5′ end (minus strand; 5′ end is to the right). The longer isoform incorporates a short ORF consisting of a start codon immediately followed by a stop codon, and a large peak of ribosome density is observed here in starved cells. Footprint density is greatly reduced at *JIP5* in starved cells, however the proximity of *NUT2* makes it difficult to assess whether the transcript architecture changes. Y-axis scales are consistent for mRNA and footprint tracks, respectively, at each locus. Footprint counts are mapped to ribosomal P-sites; RNA-seq counts are evenly apportioned across the length of each sequencing read. (B) Scatter plots of normalized counts (RPKM) in amino acid starvation experiments with- vs. without-CHX pretreatment. Ribi gene counts fall off-diagonal in starved cells. (C) Average codon composition of ribi vs non-ribi genes. Certain Asp, Lys, and Glu codons are enriched in ribi genes. (D) Genes enriched in GAA, AAA, GAU, and AAG codons have lower footprint counts in CHX-treated vs -untreated cells. While ribi genes (black) are enriched in these codons, the correlation holds for all genes.

Supplementary Figure S3.

(A) Ethanol was added to starved cells at t=0 and *NOP2* mRNA abundance was monitored over time. Percentages reflect the final concentration (vol/vol) of added ethanol in the culture. Error bars represent the standard deviation of 3 technical replicates from a single biological sample. (B) BY4741 was grown in SD in a 30 °C shaking incubator and OD was monitored over time. CHX

dissolved in ethanol or DMSO was added immediately following the 6-hour time point. CHX inhibits growth regardless of the solvent. Error bars represent the standard deviation of 3 biological replicates. (C) The prototrophic strain CEN.PK has an attenuated response to CHX in ethanol compared to the same treatment in the auxotrophic strain BY4741 (compare to Figure 3A, solid orange line). The change in *NOP2* mRNA is of similar magnitude to BY4741 treated with CHX in DMSO. Both strains have nearly identical responses to the respective vehicle treatments, which resemble an untreated control. Error bars represent the standard deviation of 3 technical replicates from a single biological sample. (D) The *NOP2* promoter was defined as the 285 bp region from the boundary of the upstream gene (*GCD10*) to the consensus *NOP2* transcript start site (2–4). Blue box, *NOP2* promoter; thin black box, 5′ UTR from (5); thick black box, coding sequence.

Supplementary Figure S4.

(A) The experiment in Figure 4C was repeated with rapamycin added to the starvation medium. In a microfluidic plate, rapamycin does not alter the localization of these transcription factors following CHX treatment. (B) Repeat of the above experiment in a flask. Cells were pelleted and re-suspended in starvation medium with (pink) or without (black) 200 nM rapamycin. After 15 min CHX was added, and after an additional 15 min cells were imaged in glass-bottom wells. All three transcription factors show increased nuclear localization in the presence of rapamycin. P-values were calculated using a two-sided t-test. (C) Ribi mRNAs still decrease following 20 min of amino acid starvation in a *dot6 tod6 stb3* triple deletion strain, though to a lesser degree than the WT strain (compare to Figure 2B).

Supplementary Table S1.

Yeast strains used in this study. MAT, mating type.

Supplementary Table S2.

Plasmids used in this study.

Supplementary Table S3.

Oligonucleotides used in this study. Non-standard bases are indicated using Integrated DNA Technologies' nomenclature (https://www.idtdna.com/). Sequences of oligos used for plasmid and strain construction are available upon request.

SUPPLEMENTARY REFERENCES

1. Brar,G.A., Yassour,M., Friedman,N., Regev,A., Ingolia,N.T. and Weissman,J.S. (2012) High-Resolution View of the Yeast Meiotic Program Revealed by Ribosome Profiling. *Science*, **335**, 552–557.

2. Arribere,J.A. and Gilbert,W.V. (2013) Roles for transcript leaders in translation and mRNA decay revealed by transcript leader sequencing. *Genome Res.*, **23**, 977–987.

3. Malabat,C., Feuerbach,F., Ma,L., Saveanu,C. and Jacquier,A. (2015) Quality control of transcription start site selection by nonsense-mediated-mRNA decay. *eLife*, **4**.

4. Park,D., Morris,A.R., Battenhouse,A. and Iyer,V.R. (2014) Simultaneous mapping of transcript ends at single-nucleotide resolution and identification of widespread promoter-associated non-coding RNA governed by TATA elements. *Nucleic Acids Res.*, **42**, 3736–3749.

5. Nagalakshmi,U., Wang,Z., Waern,K., Shou,C., Raha,D., Gerstein,M. and Snyder,M. (2008) The transcriptional landscape of the yeast genome defined by RNA sequencing. *Science*, **320**, 1344–1349.
